# Using the Sadakane Compressed Suffix Tree to Solve the All-Pairs Suffix-Prefix Problem

**DOI:** 10.1155/2014/745298

**Published:** 2014-04-16

**Authors:** Maan Haj Rachid, Qutaibah Malluhi, Mohamed Abouelhoda

**Affiliations:** ^1^KINDI Lab for Computing Research, Qatar University P.O. Box 2713, Doha, Qatar; ^2^Faculty of Engineering, Cairo University, Giza, Egypt; ^3^Center for Informatics Sciences, Nile University, Giza, Egypt

## Abstract

The all-pairs suffix-prefix matching problem is a basic problem in string processing. It has an application in the de novo genome assembly task, which is one of the major bioinformatics problems. Due to the large size of the input data, it is crucial to use fast and space efficient solutions. In this paper, we present a space-economical solution to this problem using the generalized Sadakane compressed suffix tree. Furthermore, we present a parallel algorithm to provide more speed for shared memory computers. Our sequential and parallel algorithms are optimized by exploiting features of the Sadakane compressed index data structure. Experimental results show that our solution based on the Sadakane's compressed index consumes significantly less space than the ones based on noncompressed data structures like the suffix tree and the enhanced suffix array. Our experimental results show that our parallel algorithm is efficient and scales well with increasing number of processors.

## 1. Introduction

Given a set *S* of strings *S*_1_, *S*_2_,…, *S*_*k*_, the all-pairs suffix-prefix problem (APSP) is to find the longest suffix-prefix match for each ordered pair of the set *S*. Solving this problem is a basic step in the de novo genome assembly task, where the input is a set of strings representing random fragments coming from multiple copies of the input genome. These fragments can be ordered based on suffix-prefix matching and after some postprocessing, the input genome can be reconstructed.

With the recent advances in high throughput genome sequencing technologies, the input size became very huge in terms of the number of sequences and length of fragments. This calls for both faster and memory efficient solutions for the APSP problem.

The APSP is a well-studied problem in the field of string processing. The first nonquadratic solution was introduced by Gusfield et al. [1]. Their algorithm was based on the generalized suffix tree and it takes *O*(*n* + *k* ^2^) time and linear space, where *n* is the total length of all *k* strings. Although the theoretical bounds of this algorithm are optimal, the cache performance and space consumption of the suffix tree are major bottlenecks to solve large size problems (note that the best implementation of a suffix tree consumes 20 bytes per input character [2]).

Ohlebusch and Gog [3] introduced a solution to APSP using the enhanced suffix array [4], which is an index data structure that uses only 8 bytes per input character. Their algorithm has the same complexity as that of [1]. Their algorithm has exploited interesting features of the enhanced suffix array, which has not only reduced the space consumption but also improved the cache performance and accordingly the running time. Experimental results have shown that their solution is 1.5 to 2 times faster in practice and can indeed handle large problem sizes.

In an effort to reduce the space consumption of solving the problem, Simpson and Durbin [5] used the FM index [6] to solve the problem in an indirect way as follows. The index is constructed for all strings after concatenating them in one string. The index is then queried by the reads, one by one, to find prefix-suffix matches. The time complexity of this algorithm is not as optimal as the one of [1, 3], because one examines more suffixes than the output size. (This limitation stems also from the fact that the FM index lacks structural information to run the algorithms of [1] or [3] on it.) But its space consumption is much less than that of the previous algorithms.

In this paper, we present new methods based on the compressed suffix tree of Sadakane [7] and variations of the algorithms of [1] and [3]. The compressed suffix tree is considered as a self-index data structure, because the original text is already encoded in the index in a compressed fashion and can be extracted from it; that is, there is no need to keep the original text in the memory. It is also fully functional like the uncompressed suffix tree, as it offers the typical suffix tree operations such as checking if a node is a leaf, moving to the next sibling, using a suffix link, or even performing lowest common ancestor queries. Such compressed suffix tree consumes much less space than the suffix tree and the enhanced suffix array but more space than the FM index [6], as it includes additional structural information.

To further speed up the solution of APSP, we introduce different parallelization strategies to the sequential algorithm that can be used on multiprocessor shared memory computers. Our parallelization methods exploit important features and available operations of the Sadakane's compressed suffix tree. Experimental results show that our method is efficient and scales well with the number of processors.

This paper is organized as follows. In Section 2, the data structures and the functions used in our solutions are explained. In Section 4, the two different approaches for solving APSP using Sadakane index are demonstrated.  Section 5 describes how our solutions can be parallelized, and finally we show our experimental results and conclusions in Sections 6 and 7, respectively.

## 2. Overview

### 2.1. Basic Notions

We write *S* = *S*_1_, *S*_2_,…, *S*_*k*_ to denote a set *S* of *k* strings. Each string *S*_*i*_ is defined over an ordered alphabet Σ. For strings representing genomic sequences, Σ = {*A*, *C*, *G*, *T*}, which is the standard alphabet for DNA sequence data. We write |*S*_*i*_| to denote the length of the string *S*_*i*_ and *S*_*i*_[*j*] to denote the *j*th character, where 1 ≤ *j* ≤ |*S*_*i*_|. The *j*th suffix of a string *S*_*i*_ is the substring *S*_*i*_[*j*..|*S*_*j*_|] and it is denoted by *S*_*i*_(*j*). A prefix of length *j* of a string *S*_*i*_ is the substring *S*_*i*_[1..*j*]. For two strings *S*_*i*_ and *S*_*j*_, the longest suffix-prefix match of the pair (*S*_*i*_, *S*_*j*_) is the match with the greatest *r* such that *S*_*i*_[*r*..|*S*_*i*_|] = *S*_*j*_[1..*r*].

The suffix tree of a string *S* is an index structure in which each suffix of *S* is stored as a path from the root to a leaf. Obviously many suffixes will share partial path before they end in different leaves. Accordingly, the suffix tree of a string *S* has *n* leaves and at most *n* − 1 internal nodes, where *n* = |*S*|. Suffix tree can be constructed and stored in linear time and space ([8, 9]).

### 2.2. Compressed Suffix Tree

Sadakane's compressed suffix tree [7] is composed of three major components.

**Figure 1:**
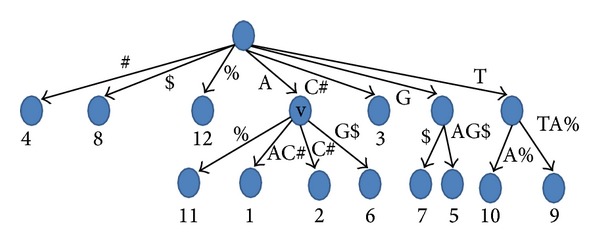
A generalized suffix tree for the string AAC#GAG$TTA%. The numbers below the leaves are the text positions for the string paths which are represented by these leaves.

- *Compressed suffix array* (CSA): the suffix array SA of a string *S* is an array of integers including the positions of the lexicographically sorted suffixes of *S*; that is, for any two integers 0 ≤ *i*′ < *i*′′ < *n*, *S*(SA[*i*′]) is lexicographically less than *S*(SA[*i*′′]). The CSA is a compressed version of SA with reduced space requirements, which can perform the traditional suffix array operations with a slight slowdown. The implementation of Sadakane suffix tree that is used in our experiments utilizes the CSA presented in [10]. It is based on wavelet tree [11] built on Burrows-Wheeler transform [12]. The space consumption of this CSA is *O*(*n*log⁡Σ).
- The* largest common prefix array* (LCP): this is an array of integers in the range 1 to *n* such that LCP [1] = 0 and LCP[*i*] is the largest common prefix between SA[*i*] and SA[*i* − 1], where 1 ≤ *i* ≤ *n*. The LCP is also compressed. In the implementation which we use, LCP is encoded using a technique described by [7] which can store LCP in only 2*n* + *O*(*n*) bits.
- The* balanced parenthesis representation* (BP): BP of a tree is generated by traversing the tree in preorder manner to produce open and closed parentheses. Initially, BP is empty. Whenever a node is visited, an open parenthesis, (, is added to BP. Whenever a node is left, a close parenthesis, ), is added to BP [13]. Accordingly, each node can be encoded using 2 bits. Since suffix tree has at most *n* − 1 internal nodes and *n* leaves, BP takes at most 4*n* bits. For example, the BP representation of the tree in Figure 1 is (()()()(()()()())()(()())(()())).

The following BP functions are used in this paper.

- Rank_()_  (BP, *i*): returns the number of occurrences of () in BP up to position *i*.
- Select_()_  (BP, *i*): returns the position of the *i*th () in BP.
- IsLeaf  (*i*): returns true if the position *i* in BP belongs to a leaf.
- Parent  (*i*): returns the position in BP for the parent node of node *v*, where *i* is the BP position of *v*.
- IsOpen  (*i*): returns true if *i* is a position for an open parenthesis in BP.
- Edge  (*i*, *d*): returns the *d*th character of the edge label of an edge pointing to node *v*, where *i* is the BP position of *v*.
- Child  (*v*, *c*): returns the position in BP for the node *i* which is the child of a node *p*, where *v* is *p*'s position in BP, and there is an edge directed from *p* towards *i* labeled by a string that starts with character *c*.

### 2.3. Constructing Generalized Suffix Tree

To solve the all-pairs suffix-prefix problem for a set of *k* strings *S*_1_, *S*_2_, *S*_3_,…, *S*_*k*_, we build a compressed suffix tree for the string resulting by concatenating all strings together in one string. Each two concatenated strings are separated by a distinct separator. These separators do not occur in any of these *k* strings. For example, if the strings are *S*_1_ = *AAC*, *S*_2_ = *GAG*, *S*_3_ = *TTA*, then we build a compressed suffix tree for the text AAC#GAG$TTA%, where #, $, and % are the distinct separators. These separators should be lexicographically smaller than any character in all strings (i.e., in the alphabet Σ). Since, in practice, there is a limitation for the number of available distinct separators, our implementation uses more than one character to encode a separator. Assuming that there are *c* distinct characters that can be used for constructing separators, log⁡_*c*_ *k* + 1 characters are needed to encode a separator in our work.

We use an array *StartPos* of size *k* to store the starting positions of the *k* strings. The size of such array is *Ω*(*k*log⁡⁡*n*) bits, where *n* is the size of the whole text. To map each position to the appropriate string, another array of size *n* is needed. This array requires space of *Ω*(*n*log⁡⁡*k*) bits. To avoid the expensive cost of this array, a binary search in the array *StartPos* can be done to retrieve the number of the string to which a specific position belongs. However, that will increase the time cost of retrieving the string identifier to *O*(log⁡⁡*k*) instead of *O*(1) time. It is easy to notice that both arrays are not necessary if the *k* strings are equal in size. In this case, we can get the string number to which a position *p* belongs by simply calculating *p*/*l*, where *l* is the length of each string.

## 3. Review of the Basic APSP Algorithm

The algorithm of [1] works as follows. First, the suffix tree is constructed for the string $\hat{S}=S_{1}{\#}_{1},S_{2},\ldots ,{\#}_{k-1},S_{k}{\#}_{k}$. The characters #_1_,…, #_*k*_ are distinct and do not exist in any of the given strings. These distinct characters are further referred to as* terminal characters* in this paper. Second, the suffix tree is traversed to create for each internal node *v* a list *L*_*v*_. The list *L*_*v*_ contains the children of *v* such that each child *c* is a leaf, and the label of the edge connecting *v* to *c* starts with a terminal character. Third, the suffix tree is traversed in a preorder fashion once again to report matches according to the following observation. Consider a leaf such that the string annotating the edges from the root to it is a complete given string *S*_*j*_. We call such leaf a* prefix leaf*. For each node *v*_*r*_ on the path from the root to the prefix leaf, the prefix-suffix matches of length |*v*_*r*_| are those between each element in *L*_*v*_*r*__ and *S*_*j*_. Accordingly, in the preorder traversal, we use *k* stacks representing the given strings and push *v*_*r*_ in stack *i* if *S*_*i*_ is in *L*_*v*_*r*__. When reaching a prefix leaf *S*_*j*_, the candidates from all parent nodes would already be in the stacks and the maximal matches are those between *S*_*j*_ and the top of each stack.

## 4. Solutions Based on the Compressed Suffix Tree

### 4.1. First Method

The compressed suffix tree supports all necessary informations to run the original Algorithm of [1] as it is. However, we observe some interesting properties that could significantly improve the performance of the algorithm with no additional time or space costs.

For filling the *L*_*v*_ lists, we will not simulate traversal of the whole tree over the compressed suffix tree. Rather, we will make use of the BP vector to move from a leaf to another using the *Select* function in constant time. Specifically, the *Select*_()_(*BP*, *i*) function will return the position of the *i*th () which represents a leaf. For each leaf and only if it has a terminal edge pointing to it (which can be checked using edge function), we add the text position of that leaf to the *L*_*v*_ list of the parent of that leaf node. In Figure 1, we give an example, where the *L* list for node *v*, which is the fourth child of the root, has one value 11 that belongs to string 3. Note that we can safely ignore the first *k*-leaves as these correspond to the terminal suffixes, where the length of each of these suffixes is a single character (one of the terminal characters).

**Figure 2:**
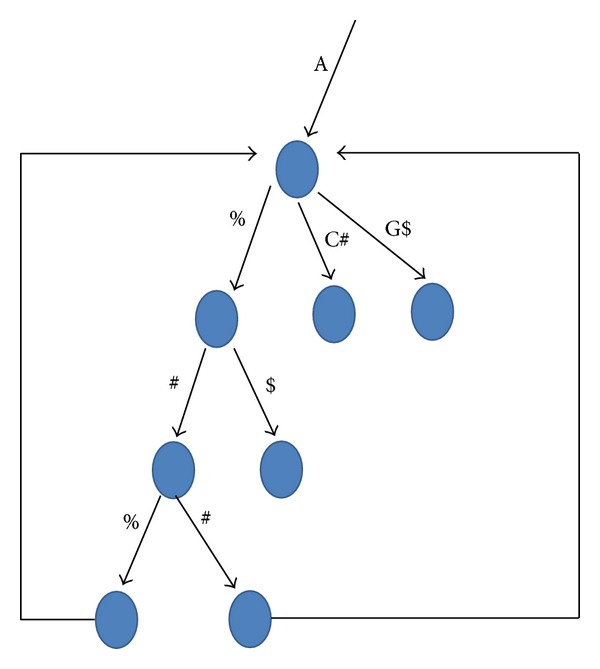
The text position for each leaf which has a terminal edge will be added to the *L* list of the closest ancestor which does not have a terminal edge pointing to it.

In the case of using more than one character to encode a distinct separator, it is possible to have an internal node to which a terminal edge is pointing (usually only leaves have this possibility). Accordingly, the text position of a terminal leaf should be added to the *L* list of its closest ancestor to which no terminal edge is pointing (see Figure 2). Let *i* be a BP position of leaf *v* and *j* is the BP position of *v*'s parent, node *z*. The pseudocode for the bottom-up traversal:

While *z* is not the root and the edge pointing to *z* is terminal,

(1) $$\begin{array}{c} j=\text{parent}\left(j\right). \end{array}$$

In the second stage, we make another scan for the BP representation from left to right, but this time we move one by one (parenthesis by parenthesis) instead of jumping from leaf to leaf. We distinguish 3 cases.

- Case 1: if the scanned node is a leaf and it is representing a starting position of a string *i*, then the top of each stack *j*, where *j* ≠ *i* and 1 ≤ *j* ≤ *k*, is the longest suffix prefix match between string *i* and string *j* (for a proof, see [1]). We can move one step ahead since the next parenthesis is the closing parenthesis of this leaf node (lines 13–19, Algorithm 1).
- Case 2: if we scan an opening parenthesis for an internal node *v*, we push each value in the list *L* of that internal node *v* to the appropriate stack (which can be found in log⁡⁡*k* time). We can determine which stack we should push the value to since this value is a text position. In Figure 1, the value 11 in *L*_*v*_ will be pushed to stack 3 (lines 20–23, Algorithm 1).
- Case 3: if we scan a closing parenthesis for an internal node *v*, we pop all values that belong to *v* from the stacks. We can easily determine which stacks to pop using *L*_*v*_ (lines 24–28, Algorithm 1).

Algorithm 1 specifies our method based on the compressed suffix tree. Lines 4–10 in Algorithm 1 compute the *L*_*v*_ lists as described above. We use *k* stacks to keep track of the leaves. The second loop (lines 11–28 in Algorithm 1) mimics a preorder traversal. All ancestors of any leaf will be visited before the leaf itself, which will guarantee that all stacks for the *k* strings will be filled before checking any leaf with a starting position. When a leaf with a starting position of a string *S*_*j*_ is reached, the top of each stack *i* will represent the longest suffix prefix match between string *i* and string *j*. Finally the closing parenthesis for any internal node will be reached after reaching all leaves in all subtrees of that internal node which guarantees the appropriate update (pop up) to all stacks. The two-dimensional array, Sol, will carry the solution at the end of the second loop.

### 4.2. Complexity Analysis

The correctness of the algorithm follows from the proof in [1]. However, in our implementation, we start the first loop with the *g*th leaf. Since *g* is incremented, we are moving from leaf to leaf until we reach the rightmost leaf. It is clear that all *L*_*v*_ lists for all internal nodes will be filled at the end of the loop.

The construction of the generalized suffix tree consumes *O*(*n*log⁡⁡*n*) time [14]. We have *n* leaves so we need *O*(*n*) time in the first loop. The second loop requires 3*n* steps since we have 2 parentheses for each leaf and 2 parentheses for each internal node, but we are avoiding the closing parenthesis of any leaf node by incrementing the counter by 1. In the second loop, we will have at most one push and one pop for each leaf so we have *O*(*n*) time complexity since all index operations which we are using (like isLeaf, Child, and Parent) have constant time [7]. The string to which the value on the top of a stack belongs is known since it is equal to the number of the stack, accordingly the time for outputting the results is *k* ^2^.

As a result, the solution requires *O*(*n*log⁡⁡*n* + *k* ^2^) time. The complexity stands even without the usage of an array to map a position to a string (this can be done by using binary search in *StartPos* array), since *n*log⁡⁡*k* is less than *n*log⁡⁡*n*.

In term of space, we need |*CSA* | +6*n* + *O*(*n*) bits to construct the tree, where |*CSA*| is the size of the compressed suffix array [7]. Since the total number of all values in all *L* lists is *O*(*n*), we need *O*(*n*log⁡⁡*n*) bits for these lists and for the stacks. The two arrays which are mentioned in the end of Section 2 require *O*(*k*log⁡⁡*n*) and *O*(*n*log⁡⁡*k*) which are both less than *O*(*n*log⁡⁡*n*). Accordingly, the solution consumes *O*(*n*log⁡⁡*n*) space.

### 4.3. Further Space Optimization

#### 4.3.1. Space Optimization 1

It is clear the *L*_*v*_ lists which are used in this method are very expensive in terms of space. One way to avoid using them is to scan the leaves once. For each leaf *e* and only if it represents a complete string *S*, we check every ancestor using the parent function until the root is reached. For each ancestor, we check every terminal edge which is coming from it. A terminal edge indicates a match between a prefix of *S* and a suffix in another string. Accordingly *L*_*v*_ lists are avoided and so are the *k* stacks. Let *ℓ* be the maximum length of all paths from the root to all leaves (which is usually less than 1500, the maximum length for a sequence). There are at most *k* terminal edge for each internal node, thereby the time consumption will be *O*(*n*log⁡*n* + *ℓk* ^2^).

#### 4.3.2. Space Optimization 2

Another variation for the first method is to keep the first stage as is, but in the second scan we check only the leaves. In this variation we will use the *L*_*v*_ lists but we will not use the stacks. If a leaf represents a complete string *S*, we check every ancestor of this leaf. Since the *L*_*v*_ lists are filled from the first stage, the values inside the *L*_*v*_ lists of the current internal node are suffix-prefix matches between *S* and suffixes from other strings. The time complexity will be the same which is *O*(*n*log⁡⁡*n* + *k* ^2^).

### 4.4. Second Method

The running time of the previous method can be improved based on the following two observations of [3].

- All the distinct characters {#_1_, #_2_,…, #_*k*_} exist in the first *k* slots in the (compressed) suffix array, because they are lexicographically less than any other character in the given strings.
- The terminal leaves (suffixes) sharing a prefix of length *ω* exist in the (compressed) suffix array before the other suffixes sharing also a prefix of length *ω* with them.

In this method, we scan the BP vector and move from leaf to leaf using the *Select* function. When a leaf is visited, we check if this leaf represents a suffix that is a prefix of the next leaf in BP. If it is, then it is pushed to the stack of the string which it belongs to. This continuous pushing is similar to creating the *L*_*v*_ lists and copying their values to the appropriate stacks. When a prefix leaf (i.e., corresponding to a whole given string) is scanned, then all pairwise prefix-suffix matches are already in the stack. An additional stack is used to keep track of the match length.  Algorithm 2 specifies how this algorithm works.

As in the first method, we ignore parentheses which belong to separators using the Child, Rank, and Select functions. We move from leaf to leaf using the Select function (lines 1–3, Algorithm 2).

To check if a leaf *i* is a prefix of the next leaf *q*, we check if *i* is a terminal leaf, and it has the same parent as the next leaf *j* in BP. If this is the case, we push the text position of *i* to the stack of *S*_1_, where *S*_1_ is the string to which the text position of *i* belongs (lines 32–42, Algorithm 2).

If the text position of *i* is a starting position of a string *S* (which can be verified using a binary search in *StartPos* array), then the top of each stack *j*, where *j* ≠ *S* and 1 ≤ *j* ≤ *k*, is the longest suffix prefix match between string *S* and string *j* (lines 7–11, Algorithm 2).

There is one exception for that; if there is a suffix in *j* which matches the string *S* and follows lexicographically the current suffix. This condition can be checked by investigating if the *i* and *j* both are terminal leaves, and they have the same parent. (lines 12–20, Algorithm 2).

The definition of the same parent depends on the number of characters used to encode the separators; if more than one character is used, then the parent of a leaf is the closest ancestor which does not have a terminal edge (Figure 2).

The second method has the same time complexity as the first method, since the construction of the tree requires *O*(*n*log⁡⁡*n*) time. For space complexity, let *ℓ* denote the maximum length of a sequence. We need at most *ℓ* of *L* lists to hold at most *n* values. Accordingly, *n*log⁡⁡*k* bits are needed for all *L* lists. We also need *n*log⁡⁡*ℓ* bits to store at most *n* values in the *k* stacks. Since *ℓ* is less than *k*, the space complexity for the second method is *O*(*n*log⁡⁡*k*), regardless of the usage of the array to map a position to a string.

## 5. Parallelizing the Algorithm

In this section, we introduce parallel versions of the above-described methods for solving the APSP problem. These versions are for shared memory multiprocessor computers. We will handle two parallelization strategies: The first, which we will call* top-down decomposition* is based on a straightforward top-down tree decomposition. The second, which we call* leaf-decomposition* is based on bottom-up decomposition.

### 5.1. Strategy 1: Top-Down Decomposition

The generalized suffix tree is divided into *P*′ subtrees occupying the highest levels of the tree. These subtrees can be processed independently in parallel. For *P* processors, we choose *P*′ = *γP*, where *γ* is a user defined parameter (We usually set it to 1.5). The roots of these subtrees are maintained in a queue. Whenever a processor is free, then one subtree is assigned to it. Each processor executes either Algorithm 1 or Algorithm 2. The *P*′ subtrees are selected by breadth-first traversal of the tree. Over the BP representation, these are selected using the child function.

For Algorithm 1, we should consider the following. Let *ω*_*r*_, where *r* ∈ [1..*P*′], denote the string annotating the edges from the root of the generalized suffix tree to the root of *r*th subtree. Let *ℓ*′ = max⁡⁡|*ω*_*r*_| is the length of the longest *ω*_*r*_ strings. Here we distinguish between two cases: (1) the minimum match length *ℓ* is larger than *ℓ*′ or (2) *ℓ* is less than *ℓ*′.

For the first case, the subtrees can be easily processed independently in parallel. The *L*_*v*_ lists on the nodes from the root of the generalized tree to the roots of the subtrees need not to be created as the respective nodes will not be processed. A processor can start executing on a subtree without filling the stacks with the values related to its ancestors.

For the second case, we will have some *L*_*v*_ lists that can be shared among two processors. For reporting the matches, there is no problem as the *L*_*v*_ lists are read only. For creating them, however, we need to use only *P*′′ < *P*′ processers, where *P*′′ is the number of *L*_*v*_ lists to be created. The stacks should be filled first with the values related to the subtree's ancestors before executing the algorithm. In our second algorithm based on [3], the *L*_*v*_ lists are not created and accordingly the above-two cases can be ignored.

**Figure 3:**
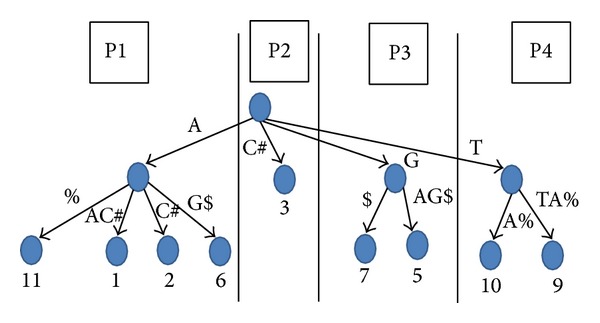
Each processor is working on one branch of the generalized suffix tree for the string AAC#GAG$TTA%. The numbers below the leaves are the text positions for the string paths which are represented by these leaves.

In Figure 3, we give a simple example where only subtrees from the top level are pushed to the queue. Assuming that 4 processors are utilized for the problem, processor 1 will work on the first child (we ignored the children which belong to #, $, and %). Processor 1 will find the answers for string 1 which is starting with an “A,” while Processor 3 which is working on the third child of the root will find the answers for string 2 which is starting with a “G.” Processor 4 which is working on the fourth child of the root will find the answers for string 3 which is starting with a “T.” Processor 2 is not going to find any answer since none of the *k* strings start with a “C.” No communication is required between processors for execution.

### 5.2. Strategy 2: Bottom-Up Decomposition

In the previous algorithm, we cannot guarantee that the subtrees are of equal sizes. Therefore, we use two tricks. First, we select *γP* subtrees, in hope of having trees of almost equal size. Second, we used a queue to keep all processors as busy as possible, which is a kind of dynamic load balancing.

Interestingly, the structure of CSA allows more robust strategy which can lead to better performance. The idea is to distribute the load equally between processors either by dividing the leaves or by dividing BP between them. Each processor starts working from the starting point of its share. It is clear that the situation is not simple; therefore, let us analyze the content of the stack for an internal node in the sequential case when the algorithm reaches that node. It can be observed that the content of each stack is whatever was pushed when visiting the node's ancestors. All other pushing work is irrelevant since it is followed by an equivalent popping before reaching the node.

Therefore, each processor can start from a specific point if its stacks are filled with the values which would be in the stacks if we reach this point while running the sequential algorithm.

To apply this concept on the first Algorithm, let us analyze the two stages for this algorithm. The first stage is relatively trivial; each leaf, if it has a terminal edge, should push its text position to the *L* list of its parent (or to the *L* list of the closest ancestor which does not have a terminal edge pointing to it). Accordingly, if leaves are distributed between processors, we will have a relatively fair deal between processors.

**Figure 4:**
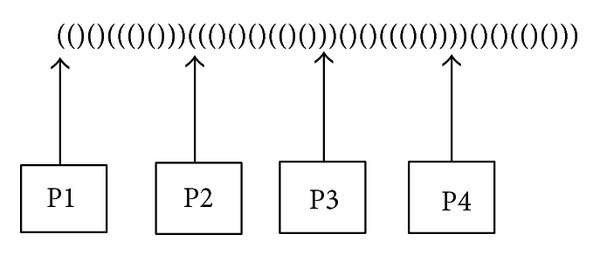
Each processor is working on its share of BP or the leaves. The stacks should be filled first for each processor before continuing with the algorithm.

In the second stage, BP vector will be divided equally between processors. Let *i* be the starting parenthesis for the processor p's share in BP (if the starting parenthesis is closing parenthesis, *i* is the first open parenthesis which comes after the starting parenthesis). The stacks of the processor p should be filled with whatever values that would be pushed when passing through the ancestors of *i* if we were working with the sequential algorithm. The parent function is recursively called for *i* until the root is reached. For each ancestor of *i*, we scan the children leaves which belongs to separators and push them in first-in-first-out way into the stacks. Each processor can then execute the algorithm on its share as if the case is sequential until the ending point of the processor's share is reached.  Figure 4 demonstrates the concept of this technique.

In the second algorithm, the *n* leaves are divided between processors using Rank and Select. Let *e* be the starting leaf for the processor p's share. Again, the Parent function is recursively called until the root is reached. For each ancestor of *i*, we scan the children leaves which belong to separators and push them in first-in-first-out way into the stacks. The algorithm then can be executed exactly as the sequential case.

### 5.3. Managing the Space Overhead

It is clear that both techniques use *k* stacks for each processer, which may appear as a problem when a large number of processors are utilized. The space issue can be solved by implementing the *k* stacks using an efficient data structure such as balanced binary search tree instead of using an array of *k* stacks. Another solution is to use the technique presented in Section 4.3, which avoids using the *k* stacks.

## 6. Experimental Results

A summary for the discussed algorithms is shown in Table 1. Experiments have been conducted to show the gain in space by comparing the space consumed by Sadakane compressed suffix tree with the space consumed by a standard pointer-based suffix tree and enhanced suffix array. We also investigated the space and time consumed in the overlap stage of a recent string graph-based sequence assembler called SGA [15]. SGA is a software pipeline for the de novo assembly of sequencing readsets. The experiments also evaluate the scalability of the proposed parallel technique and compare it with the traditional ways to parallelize a suffix tree.

To compare our work with previously presented solutions, we downloaded a solution for all-pairs suffix-prefix problem using Kurtz implementation for a standard suffix tree and the implementation presented by Ohlebusch and Gog for an enhanced suffix array from http://www.uni-ulm.de/fileadmin/website_uni_ulm/iui.inst.190/Forschung/Projekte/seqana/all_pairs_suffix_prefix_problem.tar.gz.

SGA can be downloaded from http://github.com/jts/sga/zipball/master.

In our experiments, the implementation of Sadakane compressed tree presented by Välimäki et al. ([14, 16]) is used. This implementation is tested in the work of Gog [17]. It is available at http://www.cs.helsinki.fi/group/suds/cst/cst_v_1_0.tar.gz. We used it to write two C++ solutions for the APSP problem, compiled with openMP flag to support multithreading. Our implementation is available for download at http://confluence.qu.edu.qa/download/attachments/9240580/SADAApsp.zip.

### 6.1. Experimental Setup

In our solutions, the user can specify the parallel technique from the command line. For each algorithm, we implement both bottom-up and top-down parallelizing techniques. The number of threads can also be given as a parameter. If the top-down technique is used, the number of threads should be 4^*b*^, where *b* ≥ 0. Another parameter is the minimal length to be accepted as a suffix-prefix match between two strings. Accordingly if the length of the longest suffix-prefix match between any two strings is less than the minimal length, then 0 is reported.

In our solution, all strings are concatenated together in one text to build a generalized suffix tree. To overcome the limitation of the number of separators, we used 3 characters to encode enough separators for *k* ≤ 200^3^ = 8000000 strings (assuming that a character can encode around 200 separators). Our experiments for the sequential test were run on machines having Linux Ubuntu version 11.10, 32-bit with 3 GB RAM, Intel 2.67 GHZ CPU, and 250 GB hard disk.

Our results are obtained by running against randomly generated as well as real data. The random data were generated by a program that outputs random *k* strings with random lengths, but with a total length of *n*, where *n* and *k* are specified by the user. The random numbers were drawn from a uniform distribution. The real data, which are the complete EST database of* C. elegans*, are downloaded from: http://www.uni-ulm.de/in/theo/research/seqana. The size of the total length for the real data is 167,369,577 bytes with *k* = 334,465 strings. We use the average of 5 readings for each data point.  Table 2 describes our data sets.

To test our parallel technique, we used Amazon Web services (AWS) to obtain an instance with 16 cores. Our parallel implementation uses the OpenMP library.

### 6.2. Experimental Evaluation

**Figure 5:**
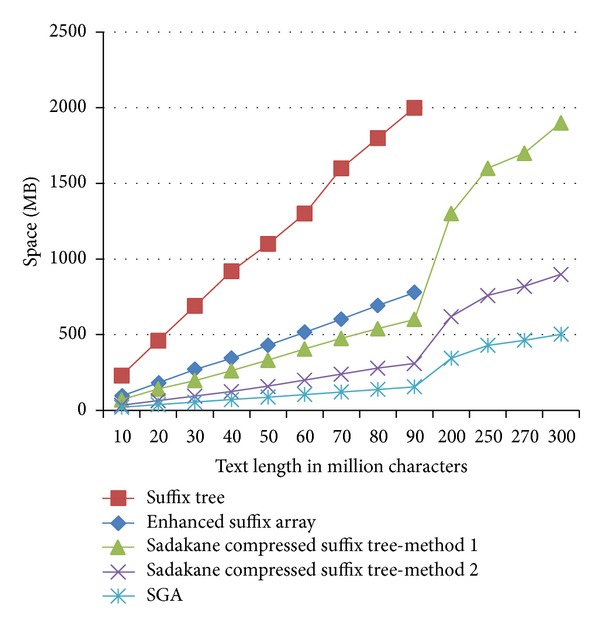
Comparison of space requirements for the three structures (standard suffix tree, enhanced suffix array, and Sadakane compressed suffix tree methods 1 and 2). In addition, the space consumed by the overlap stage in SGA is also shown. The used minimal length is 15.

Experimental results demonstrate that the first method uses around one-third of the space used by a standard pointer-based suffix tree to solve the same problem, while the second method uses less than one-fifth of the space consumed by a standard suffix tree (see Figure 5). We interpret the difference in space consumption between the two methods as a consequence of the difference in space complexity and the difference in number of the *L* lists that are used in the two methods.

**Figure 6:**
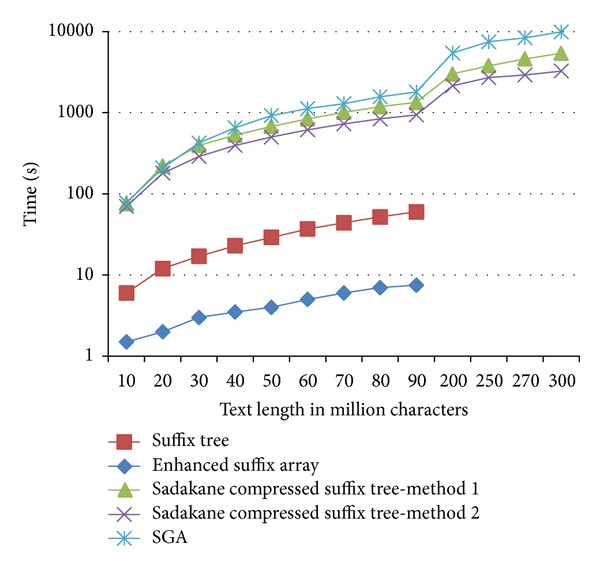
Comparison of time requirements for the three structures (standard suffix tree, enhanced suffix array, and Sadakane compressed suffix tree methods 1 and 2): we could not run the code to build a standard suffix tree for a text with a size which is bigger than 80 MB or an enhanced suffix tree for a text with a size more than 90 MB. The time consumed by SGA in overlap stage is also shown. The used minimal length is 15.

However, this gain in space has some consequences.  Figure 6 demonstrates an obvious slowdown of our solution, which is an expected price to pay as a result of using a compressed data structure. Nevertheless, we were able to run our tests using Sadakane compressed suffix tree with a text of a length that is larger than 300 MB, while the maximum size of text which we could run our test on, using a standard suffix tree or an enhanced suffix array, was 90 MB. Therefore, our solution offers better utilization of space resources and allows the user to run larger jobs without the need to upgrade hardware. In addition, our solutions overcome the practical total number of strings limitation (i.e., *k* is not limited to 200).

Despite the impressive space consumption of SGA, our solutions consume less time than SGA. In addition, the performance of SGA depends dramatically on two factors: the maximum length of a sequence and the minimal length of a match. Since the time complexity of our solution depends on *n* where *n* is the total length of all strings, both factors do not affect the performance in our solutions. Our results show that SGA fails to create its index for the overlap stage when *ℓ* ≥ 4000, where *ℓ* is the maximum length of a sequence.

**Figure 7:**
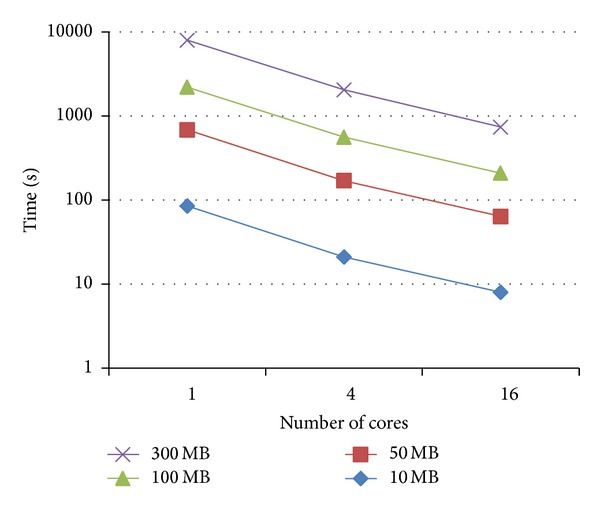
Time requirements for solving APSP for random data with four different text lengths (10 MB, 50 MB, 100 MB and 300 MB), using top-down technique with 1, 4, and 16 cores.

**Figure 8:**
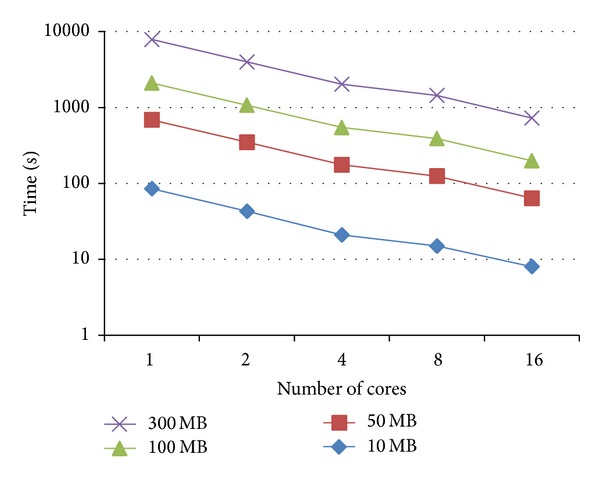
Time requirements for solving APSP for random data with four different text lengths (10 MB, 50 MB, 100 MB, and 300 MB), using bottom-up technique and various number of cores.

The parallel tests show the following: with random data, all techniques take around 24–26% and 9–11% of the time required by the sequential test, with 4 and 16 cores, respectively. Figures 7 and 8 show that both techniques demonstrate good scalability. No significant difference in performance is observed between the two methods.

**Figure 9:**
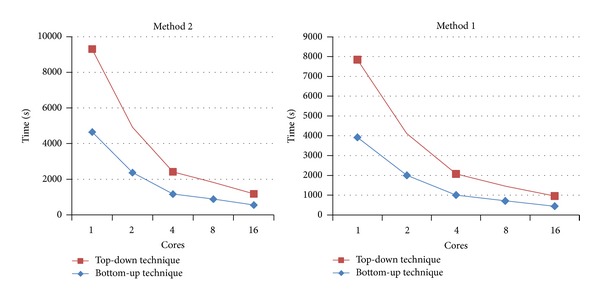
Time requirements for solving APSP for real data (167,369,577 bytes), for both methods using top-down and bottom-up techniques.

With real data, the bottom-up technique achieves a speedup of 11–13% compared with the performance of the top-down technique. It is also noticable that the second method [3] consumes with real data more time than the first method [1] (Figure 9). This is due to the fact that the real data has a considerable number of strings which are suffixes of others, which causes the special case (exception) in method 2 to occur frequently.

## 7. Conclusion

This paper provides two solutions for the all-pairs suffix-prefix problem using Sadakane compressed suffix tree, which reduce the expensive cost of suffix tree in term of space. In spite of significant slowdown in performance, it is clear that the proposed solutions may be preferred when dealing with huge sizes of data because of its modest space requirement. To reduce the performance overhead, the paper presented static and new dynamic techniques to parallelize the proposed solutions. The bottom-up technique performs more efficiently when real data is used, while both techniques perform equally with random data. The presented solutions are not limited to cases with a small number of strings. SGA is superior in terms of space, but it consumes more time than the presented solutions and it does not handle sequences which have large lengths. The paper has demonstrated that it is beneficial to use an enhanced suffix array to solve APSP. It could be worthwhile to explore solving the problem using a compressed suffix array and a compressed largest common prefix (LCP) array by adapting the algorithm presented by Ohlebusch and Gog, which makes the topic a good subject for future study.

## Figures and Tables

**Algorithm 1:**
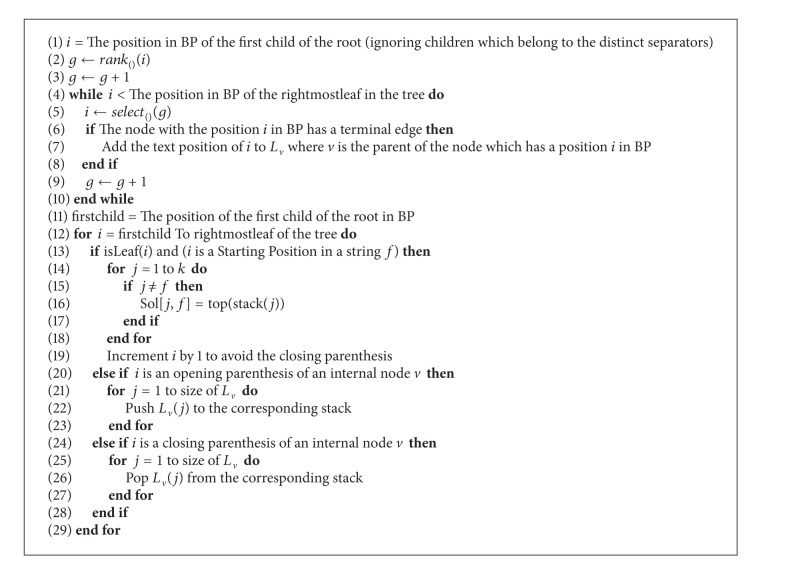
First method.

**Algorithm 2:**
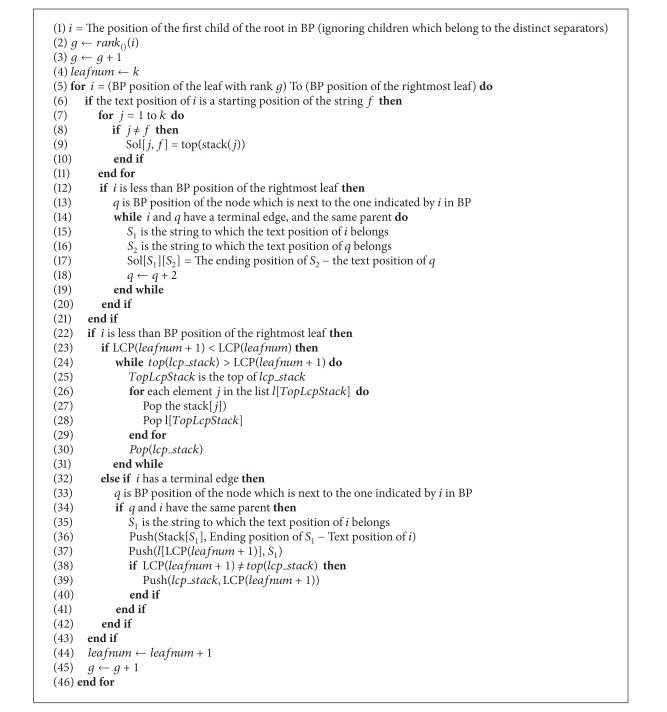
Second method.

**Table 1:** Comparison between the two methods in term of time and space complexity. Time and space used for output are ignored.

Algorithm	Used data structures	Time complexity	Space complexity
First method	BP and CSA	*O*(*n*log⁡*n*)	*O*(*n*log⁡*n*)
Second method	BP, LCP and CSA	*O*(*n*log⁡*n*)	*O*(*n*log⁡*k*)

**Table 2:** Data sets used in experiments. Sizes in megabytes.

Data Set	Type	Size	Number of strings
Generated by a program	Random data	10–300	100,000
EST of *C. elegans*	Real data	167	334,465
